# Winnie Byanyima answers questions about the impact of the COVID-19 pandemic on the UNAIDS’ Fast Track targets for 2020

**DOI:** 10.1038/s41467-020-20111-5

**Published:** 2020-12-01

## Abstract

Winnie Byanyima is the Executive Director of UNAIDS and leads the United Nations’ efforts to end the AIDS epidemic by 2030. She is also a longstanding champion of social justice and gender equality having led Uganda’s first parliamentary women’s caucus where she championed gender equality provisions during her 11 years as an elected member of the Ugandan parliament. To mark World AIDS Day 2020, *Nature Communications* interviewed Winnie about how the COVID-19 pandemic has impacted the UNAIDS Fast Track targets, the impact of both epidemics on women around the world, and what is next in the fight against HIV.

**Figure:**
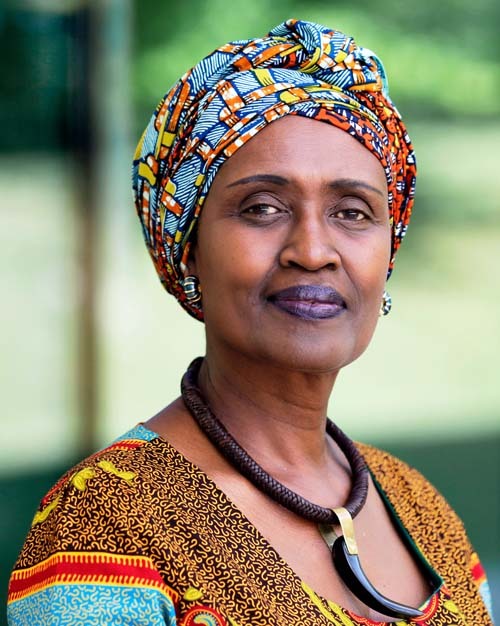
Winnie Byanyima

1. The UNAIDS’ Fast Track targets for 2020, known as the 90-90-90, aimed to achieve the following goal: 90% of people living with HIV would know their status; 90% would be under antiretroviral treatment, of whom 90% would be virally suppressed. You have previously stated that these targets are unlikely to be met in 2020. How has the Covid-19 pandemic impacted our ability to achieve these goals?

The 90–90–90 targets have been greatly successful at focusing global attention on the importance of diagnosing people living with HIV as soon as possible after their acquisition of the virus, of ensuring that diagnoses are quickly and systematically followed by the initiation of antiretroviral therapy, and then supporting individuals to be retained in treatment and achieve durable suppression of the virus to undetectable levels. Several countries across a range of epidemic and economic situations have achieved these targets. Globally, 81% of people living with HIV knew their HIV status, and among those people, 82% were on treatment—more than 25 million people—and 88% of those on treatment had achieved viral suppression at the end of 2019.

That is great progress! But we are still far behind on our impact targets, especially on reducing new HIV infections. One problem is that 90–90–90 has come to mean ‘Fast-Track’, but 90–90–90 is just one of 10 major targets agreed by the United Nations General Assembly in 2016. Progress towards the other targets—such as eliminating vertical transmission and ensuring that children are diagnosed and on treatment, condom promotion, harm reduction, pre-exposure prophylaxis and voluntary medical male circumcision—has been much slower. Sufficient uptake of these services is impossible when women and girls are still denied the education, economic independence and voice they need to protect themselves from HIV, and too many countries systemically and aggressively harass and punish populations at high risk of HIV infection, such as gay men, sex workers and people who inject drugs. A strong combination of biomedical, behavioural and structural approaches is needed to defeat this epidemic. That combination requires sufficient investment. But we are not only failing to reach the investment target set for 2020, the funding gap for the HIV response is widening.

All of this was before COVID-19, which is indeed having an impact on the HIV response, and specifically on efforts to reach the 90–90–90 targets. Anyone who has visited a health facility in recent months can understand the issue. Chronically under-resourced health systems are being overwhelmed by an unprecedented health crisis. As they react to the challenge, they must triage. Less urgent services slow or even stop, and new ways must be found to deliver urgently needed care within the context of lockdowns, curfews and physical distancing. UNAIDS has been monitoring this situation, and also working with our international partners to provide guidance and support as countries work through these challenges. Among countries that are reporting monthly to UNAIDS, most have been able to continue providing antiretroviral therapy to their *existing* patients, in part due to the acceleration of differentiated models of care such community-based service delivery and multi-month dispensing of medicines. However, in many countries, the number of people being diagnosed and enrolled in treatment have declined precipitously. We are not getting many new patients. Access to viral load testing has also been disrupted. If rates of new HIV infections in 2020 are similar to previous years and these service disruptions continue, we could see the percentages of people living with HIV who are undiagnosed, not on treatment and not virally suppressed increase instead of decrease. How long this lasts depends a lot on countries’ abilities to strengthen and adapt their health systems, and on the world to collaborate together on vaccines and other biomedical innovations, as well as the behavioural and structural changes needed to bring both the COVID-19 and HIV pandemics under control.

2. The recent results of the HIV vaccine trial HVTN 702 showed that the vaccine was ineffective in preventing HIV infection and so has been discontinued. How has the recent Covid-19 pandemic impacted research into the development and testing of new and existing vaccine candidates for treating HIV?

An effective, accessible, affordable and equitable vaccine for HIV remains a massively important goal. It is also an example of where the infrastructure and experience of the HIV response are being leveraged to fight COVID-19. The huge network of HIV trial sites across the world is being used as the foundation for a new major Covid-19 Vaccine Network. The network is essentially jump-starting COVID-19 vaccine trials.

COVID-19 is also bringing many challenges to two ongoing large-scale trials for an HIV vaccine: the Imbokodo trial among women in southern African, and the Mosaico trial among populations of gay men, other men who have sex with men, and transgender people in the Americas and Europe. The partners running these trials have put in place safety measures across all trial sites to prevent the spread of COVID-19 and to ensure the safety of study participants, investigators and site staff. These measures include enhanced use of personal protective equipment and the use of telephone and virtual methods for trial participant screening and follow up. The Mosaico trial was forced by COVID-19 to temporarily halt certain clinical trial activities such as screening and new enrolments. Site-by-site risk assessments have been conducted to determine whether it is safe to resume normal clinical study activities, and many such activities resumed in June.

So, the good news is that efforts to develop an HIV vaccine are continuing despite the challenge of COVID-19, and this includes several other vaccine candidates that are in earlier stages of development. The situation also underscores the complexity of vaccine development.

3. The “HIV Policy Lab” is a tool to track global policies on HIV pandemic responses, allowing for transparency, comparisons and support of governmental initiatives, how do you anticipate this being used for HIV policies more globally? Are there potential implications for emerging pandemics for unrelated diseases, like COVID-19?

The Global AIDS Monitoring system managed by UNAIDS compiles a wealth of data on HIV-related laws and policies. To maximize use of these data, UNAIDS has joined forces with Georgetown University, the O’Neill Institute for National and Global Health Law, the Global Network of People Living with HIV and Talus Analytics to launch the HIV Policy Lab. There is a deep and growing body of evidence that shows how legal and policy environments can either facilitate or block access to HIV services. Parliaments, governments and the courts can either mitigate or aggravate societal marginalization of people living with HIV and people at risk of HIV infection. This is why all countries agreed within the United Nations General Assembly’s 2016 Political Declaration on Ending AIDS to create enabling legal, social and policy frameworks in order to eliminate HIV-related stigma, discrimination and violence—including all forms of violence faced by women and girls

The HIV Policy Lab is a tool designed to leverage progress on this commitment by presenting data on HIV-related laws and policies in a format that is understandable to everyone—not just bureaucrats and scientists. It supports efforts by civil society groups in countries to advocate for legal and policy change and hold their governments to account. Governments themselves can use the tool to guide their efforts to fulfil their commitment, and to benchmark their progress against countries in their regions or with similar economic and epidemiological situations. Regional and global partners can use it to identify good practices and amplify those experiences to push forward the global agenda on reforming laws and policies so they enable the building of robust responses to HIV.

The HIV Policy Lab is also the latest piece of a multisectoral monitoring and reporting network that is one of the great strengths of the global AIDS response. Efforts to fight COVID-19 and responses to other emerging pandemics can learn from and build on this experience, which recognizes that health is deeply connected to social protection, education, rights and social justice.

4. You are a longstanding champion for gender equality: what can you tell us about the dual impact of HIV and Covid-19 on women around the world?

COVID-19 has exposed the deeply embedded gender inequalities that enable intimate partner violence and the educational and economic subjugation of women and girls across the world, which in turn negatively impact their ability to protect themselves from HIV infection, access health care services and claim their sexual and reproductive rights.

As was also the case during the Ebola crisis, school closures implemented to fight the spread of COVID-19 are hitting girls the hardest. If specific protective measures are not taken, closing schools leads to increases in gender-based violence, teenage pregnancies and child marriage. Some girls who had their educations disrupted by the pandemic will never go back to school, reinforcing the vicious cycle of poor education and skills, insecure jobs in the informal economy, increased vulnerability and less control over their futures.

Supply and service disruptions to health services are also hitting women hardest. This situation is especially dire for migrants and women living in conflict zones or refugee camps—places where there is often a lack of clean water and sanitation, limited ability to practice physical distancing, and a paucity of PPE and relief supplies. COVID-19 lockdowns and restrictions have also been insidiously used against women who do not neatly fit within conventional social norms, including the harassment and detention of sex workers, lesbians, women who use drugs and migrant workers.

The COVID-19 pandemic has also accelerated the pandemic of violence against women. Our colleagues at UN Women have compiled reports of women and girls living in abusive situations who have been literally trapped in violent households during lockdown. Seeking support or shelter in these situations is difficult under normal conditions. Now it is even worse. There are reports of shelters for violence survivors being closed or transformed into homeless shelters, mobile clinics and counselling services being cancelled.

This erosion of hard-won gains towards gender equality has very real impacts on efforts to fight the HIV pandemic. Additional years of secondary schooling have been correlated with reduced incidence of HIV among adolescents and young people. Cash transfers that keep girls in school and economic empowerment activities have been shown to reduce transactional sex and the risk of acquiring HIV. COVID-19 is disrupting these efforts. Intimate partner violence has been shown to increase HIV risk in settings with HIV prevalence, and there is solid evidence that intimate partner violence blocks women living with HIV from accessing and sustaining the treatment they need, which erodes health outcomes. COVID-19 has caused disruptions in access to sexual and reproductive health services and STI/HIV testing and treatment initiation.

5. You have also been a vocal proponent for a ‘People’s Vaccine’ for coronavirus, which ties in with the theme of 2020 World Aids Day of “Global solidarity, shared responsibility”. How will this be implemented around the world?

In the fight against COVID-19 we have been witnessing vaccine nationalism over the past few months. Rich nations representing just 13% of the world’s population have bought up more than half of the promised doses of leading COVID-19 vaccine candidates.

This is short-sighted. COVID-19 does not recognize nationalities and it does not respect borders. The pandemic is a stark reminder of how connected and interlinked our world is. As UN Secretary-General Antonio Guterres has said, “No one is safe, until all of us are safe”. My health depends on your health.

We need a People’s Vaccine, as opposed to a profit vaccine, one that is available for all, everywhere, free of charge at the point of use. At its core is the principle that health is a human right—not a privilege. It must never depend on the money in your pocket, the colour of your skin, or the country you were born in. This is the painful lesson we have learned from the early days of the AIDS response when millions of lives were needlessly lost as the high cost of life-saving treatment kept it out of reach for the poorest and the global South.

The People’s Vaccine alliance calls for global solidarity and cooperation, which is the only way we will defeat the COVID-19 pandemic. In order to achieve this, we need monopoly free vaccines. Public funding for research and development must be conditional on research institutions and pharmaceutical companies sharing all information, data, biological material, know-how and intellectual property rights. WHO’s COVID-19 Technology Access Pool provides the mechanism for such sharing. Pricing must be transparent and based on the cost of research, development and manufacturing, as well as taking into account any public funding provided. And we need fair allocation of the vaccine which prioritizes health workers and other at-risk groups in all countries. Rich countries should contribute the doses they have secured to global mechanisms such as Covax, and allocation between and within countries should be based on need and not ability to pay.

The People’s Vaccine alliance is a diverse, global and growing movement, which I am proud to be co-leading. It includes representatives of the HIV community to ensure we learn from the past. One such lesson is that placing affected communities at the centre of a pandemic response is crucial: in governance and planning, community service delivery and monitoring and accountability.

The People’s Vaccine has been endorsed by over 150 leaders and advocates globally including the Chairperson of the African Union Commission, the Presidents of Ghana, Nigeria, Senegal and South Africa, and the Prime Minister of Pakistan. We expect more to join, in the spirit of “global solidarity, shared responsibility”, the theme of the 2020 World AIDS Day.

6. What do you anticipate as being the priorities for UNAIDS for the next decade?

A new five-year strategy for the global AIDS response is currently being development through a process led by the UNAIDS board and engaging a broad range of stakeholders, including networks of people living with HIV and people most at risk of infection. I hesitate to jump in front of that process and declare what our priorities will be. But I can share a few of the themes that are emerging from this process.

One is a very strong call to place the people and communities we serve at the centre of the strategy. Infants exposed to HIV, children and adults living with HIV and people at high risk of HIV infection are our priorities. Community leadership has been at the heart of so many breakthroughs in the HIV response, from making antiretroviral medicines affordable and widely available to people living with HIV, to finding creative ways to provide a range of HIV prevention, testing and care services to the most marginalized. This community leadership has also been central to efforts to continue the provision of HIV services during the COVID-19 crisis. Community leadership needs to be codified into the strategy for the next five years of the HIV response.

Second, those communities require the support of robust public health systems that have the capacity required to provide the health services that are at the core of the HIV response, including HIV testing, antiretroviral therapy, pre-exposure prophylaxis, family planning and other sexual and reproductive health services, and screening and treatment for sexually transmitted infections, tuberculosis and viral hepatitis. Together, health systems and communities can delivery these services through differentiated means, from traditional clinical settings to community-based services that respond to the needs of the often-marginalized populations who are most at risk of HIV.

Third, the next strategy needs to pay far greater attention to societal, legal and policy impediments. Progress on stigma and discrimination—whether it is related to HIV status or a person’s sexual preference or a person’s gender or substance use—is too slow. We must do better. Highly motivated community activists and health workers can achieve only so much when the people they need to reach are being beaten by intimate partners or harassed by police. Growing recognition of the rights of LGBTIQ has gone hand in hand with increases in HIV service access for gay men, other men who have sex with men and transgender people. We can make similar gains by recognizing sex work as work and by ending the persecution of people who use drugs within the decades-long failure that is the War on Drugs. And as I mentioned earlier, women and girls must be given equal access to education and employment so they can build economic independence, and they must be able to claim their sexual and reproductive health rights and live free of violence both in their homes and in their communities. And people living with HIV should feel no shame when visiting a health facility for testing or treatment, and they should not face any restrictions to education, work or travel. We must reaffirm the vision of zero discrimination to achieve the sharp reductions in HIV infections that have been promised within the Sustainable Development Goals.

As we grapple with a new killer pandemic, we must also act decisively against existing health threats. To do otherwise is to risk being completely overwhelmed. Years from now, we do not want to look back on 2020 and say that the world once again refused to heed a clear warning to pay much greater attention to the fulfilment of the right to health.

